# Multiplexed Paper Microfluidics for Titration and Detection of Ingredients in Beverages

**DOI:** 10.3390/s19061286

**Published:** 2019-03-14

**Authors:** Alisha Prasad, Tiffany Tran, Manas Ranjan Gartia

**Affiliations:** 1 Department of Mechanical and Industrial Engineering, Louisiana State University, Baton Rouge, LA 70803, USA; aprasa9@lsu.edu; 2 St. Jospeh’s Academy, 3015 Broussard St, Baton Rouge, LA 70808, USA; 2021852@sjabr.org

**Keywords:** food safety and monitoring, microfluidic paper-based analytical device, multiplexed sensor, packaged fruit juice testing, ready-made test strips, on-spot routine analysis

## Abstract

Food safety and access to systematic approaches for ensuring detection of food hazards is an important issue in most developing countries. With the arrival of paper-based analytical devices (µPADs) as a promising, rapid, easy-to-use, and low-cost analytical tool, we demonstrated a simple microfluidic-based titration study for the analysis of packaged fruit juices. Similar, to the titration experiments using traditional glassware in chemistry laboratories, in this study the titration experiments were developed using paper microfluidics for the analysis of several analytes such as pH, vitamin C, sugars, and preservatives present in the packaged fruit juices. The allergen found commonly in dairy based mixtures and the non-pathogenic biochemical component responsible for food spoilage in cider based fruit juices were also determined. The results obtained using paper microfluidics were compared with those obtained using a conventional spectrophotometric technique. Finally, a paper microfluidics based multiplexed sensor was developed for the analysis of common nutritional ingredients, an allergen, and a non-pathogenic byproduct present in packaged fruit juices on a single platform. Overall, the results presented in this study reveal that the proposed paper microfluidic assisted colorimetric multiplexed sensor offers a quick and reliable tool for on-spot routine analysis for food safety applications.

## 1. Introduction

Consuming food with a standard nutritional value, quality, and safety is a prerequisite and a basic need for a good and healthy life [1]. With the increase in consumer demand and the limited shelf-life of the packaged products, it is challenging for the food industry to maintain such standards at sustainable costs [2]. Although, the USA-FDA (Food and Drug Administration) agency has established stringent food safety systems, several foodborne illnesses have been reported every year. Recently, US Public Health Service reported food poisoning due to *Salmonella* after consumption of beverage from Burien Fresh Smoothies during early August, 2018 [3]. Most of these food regulatory agencies rely on traditional analytical methods such as high-performance liquid chromatography (HPLC), gas chromatography coupled mass spectrometry (GC/MS), and enzyme-linked immunosorbent assay (ELISA) for food safety assessment [4]. Although these techniques are effective, it requires large sample volumes, reagents, heavy machinery, and trained personnel to perform the tests and report the results [5]. In order to solve this issue, more practical approaches for food monitoring has also been reported. For example, Adkins et al. used a paper-based analytical device (µPAD) integrated with micro wire electrodes for electrochemical detection of glucose in commercially sold carbonated beverages [6]. The same research group recently published a review article elaborating the use of electrochemical biosensors for food analysis [7]. In another study, Cuartero et al. demonstrated an electrochemical-based µPAD system utilizing cyclic voltammetry to detect chloride, bromide and iodide ions in water and food samples [8]. In another study, Jana et al. developed a μPAD assisted device for colorimetric detection of foodborne pathogens such as *E. coli*, *Salmonella typhimurium*, and *L. monocytogenes* species found commonly in staple food samples [9]. Scientists have also utilized paper-based lateral flow assays for detecting organophosphate pesticides with a detection limit of ~1 nM within 5 min. in various food and beverages [10]. Most of the literature on food safety is reported on detection or identification of pathogens (e.g., *E.-coli*, *salmonella*), pesticides (e.g., bendiocarb, carbaryl, paraoxon, malathion), herbicides (e.g., methyl viologen), heavy metal ions (Pb, Cd, Cu, Cr, Hg, Ni), or food additives, coloring and sweetening agents. 

Several multiplexed sensor strategies based on electrochemical approach have also been reported for detection of food borne pathogens [11]. Surface plasmon based (SPR) nano-biosensing technology have also been applied to detect common food allergens such as milk casein, nut allergens, soy source, and eggs [12]. Researchers have also utilized microarray technologies for detection of mycotoxins in food [13]. Multiplex paper microfluidic based approaches have been demonstrated mostly for clinical diagnosis. Although, these studies are related to food safety and assessment, literature for on-spot multiplexed analysis to help knowing the components present in the beverages or packaged fruit drinks are scarce. 

µPADs has recently drawn huge attention as a promising analytical tool as it is low-cost, easy-to-perform, and relatively fast [14]. This approach uses patterning of porous filter paper using hydrophobic wax to create hydrophilic channels where reagents can be transported utilizing the capillary action without needing any external devices. It is termed as ‘paper microfluidics’ since paper is used as a supporting substrate to create the devices which allows to perform various chemistry experiments with only micro volume of fluids [15]. Though, µPADs were introduced a decade ago, and several studies has been reported ever since, the development of multiplexed sensors for food safety is still in its infancy [16,17]. With the aim to explore and apply the analytical chemistry knowledge for real world applications, a simple and engaging paper microfluidic titration experiment is demonstrated. Herein, we demonstrated a paper microfluidic based titration study for the identification of common analytes present in fruit juices. In lab scale titration, a known concentration of titrant from a burette is flushed into another solution of unknown concentration until the reaction reaches the endpoint, which is generally indicated by a color change. Several types of titration, for example, acid-base and oxidation-reduction titrations are well established in chemistry labs, however, its demonstration in real-world settings for food safety applications is still lacking due to the requirement of expensive consumables, and large titrant volume. Paper microfluidics on the other hand, provides rapid on-spot analysis of several analytes with visible color change for easy perception and interpretation [18,19]. 

Since, the nutritional content of whole fruit remains constant, packaged fruit juice was used for this study. It is also important to note that the content of the same class of orange juice differed with the brands. Furthermore, the popular labels for example “100% pure”, or “added flavors”, or “fruit favored milk shakes” etc., ideally attract customers but are often misleading. The so-called 100% pure packaged juices are mostly extracted and stored in huge oxygen-depleted tanks for up to 10–12 months before it is packaged for sale. This fermentation method enhances the colors, but then it loses its flavor, and hence such fruit juices are blended with preservatives, sweeteners, and fruit fragrances and further labelled as “added flavors”. Similarly, fruit flavored milk shakes, is presumed to contain fruits blended with dairy milk, but the final processed pack mostly contains milk artificially flavored with the fruity fragrances [20].

In the present study, we used paper microfluidics and developed a colorimetric multiplexed sensor for the analysis of the contents present in packaged fruit juices which could be utilized on a routine basis for food safety applications. 

## 2. Materials and Methods

### 2.1. Materials and Chemicals 

Chemicals such as ascorbic acid, sodium nitrite, glucose, citric acid, potassium iodide, potassium iodate were obtained from VWR Life Science (Randor, PA, USA). The pH indicators such as phenol red, bromophenol blue, chlorophenol red, and bromocrescol green were purchased from BDH-VWR Analytical (Randor, PA, USA). Enzymes such as glucose oxidase and horseradish peroxidase were procured from Sigma Aldrich (St. Louis, MO, USA). Indicators such as Griess reagent, starch, phenolphthalein, and guaiacol were obtained from VWR Life Science (Randor, PA, USA). Lactaid capsules were purchased from Amazon, USA. Whatman filter paper No.1 (11 µm pore diameter) was provided from Bio rad Laboratories, USA. Distilled water used for experimentation was obtained from a Milli-Q water purifier.

### 2.2. Preparation of Paper Microfluidics for Titration Experiments

**Figure 1:**
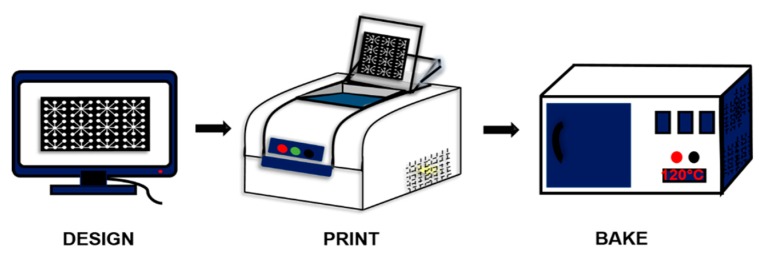
Schematic representation of the fabrication process of paper microfluidic devices. Step1: AutoCAD Design; Step2: Wax Printing on Whatman Paper Grade No.1; and Step 3: Bake at 120 °C for 2 min.

For demonstration of paper based titration study, several channels were designed using AutoCAD 2016, printed (Xerox ColorQube 8580/N Wax Printer, Xerox Corporation, Norwalk, CT, USA), and baked (for 2 min @ 120 °C) [21] (Quincy Lab 20GC Gravity Lab, Chicago, IL, USA) (Figure 1).

**Figure 2:**
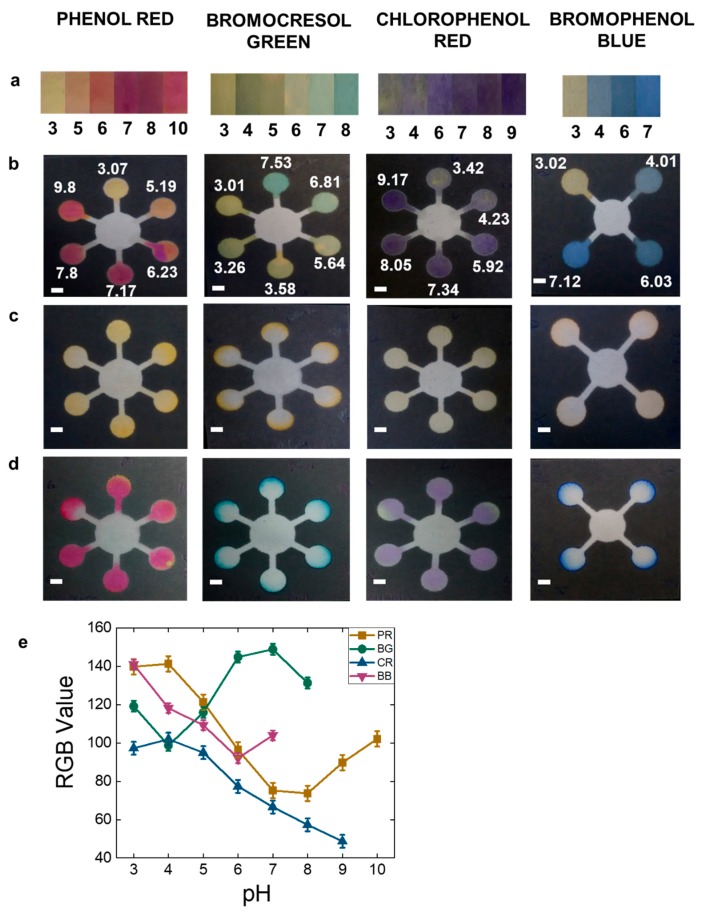
pH testing using different dyes. (**a**) pH color bar chart; (**b**) standards at different pH; (**c**,**d**) Test samples: (**c**) citric acid (pH: 2.97), (**d**) nitrite (pH: 8.64) (Scale bar: 6 mm), (**e**) RGB value corresponding to pH. PR: Phenol red, BG: Bromocresol green, CR: Chlorophenol red, BB: Bromophenol blue.

**Figure 3:**
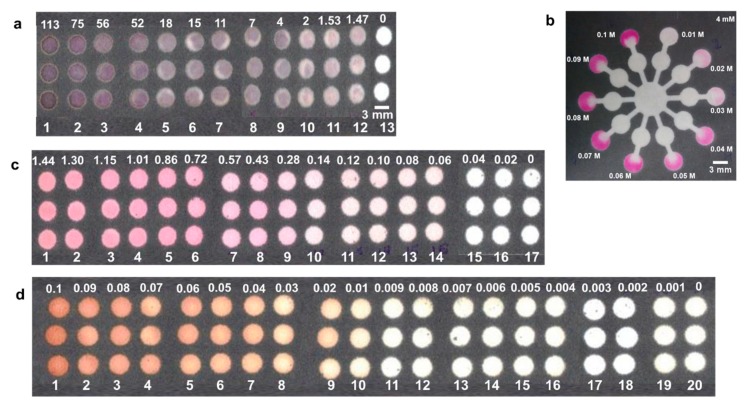

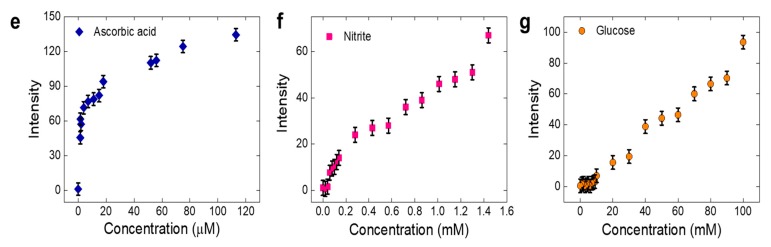
Demonstration of titration on paper microfluidics. (**a**) Ascorbic acid (concentrations are in µM); (**b**) citric acid; (**c**) nitrite (concentrations are in mM); (**d**) glucose (concentrations are in M); (**e**) Intensity Profile of a; (**f**) Intensity Profile of c; (**g**) Intensity Profile of d. (Scale bar: 3 mm).

A four-star and six-star channel design was used to demonstrate pH testing using four common pH indicators namely, phenol red, bromophenol blue, chlorophenol red, and bromocrescol green to implement on paper microfluidics (see Supplementary Information for the detailed protocol and mechanisms of action). All these dyes are yellow at pH 3 and changes to a different color with increasing pH. One drop of 1.5 μL of these pH indicators were loaded on the pH sensing zones (outer labelled circles) (Figure 2) and dried at room temperature. After drying another drop of 1.5 μL of the different pH indicators solutions were added on the same spot for producing brighter color palettes. All the experiments were performed in triplicates at room temperature of ~25 °C with relative humidity of 50%. The pH test was further evaluated by introduction of 10 μL of citric acid (Figure 2c) and sodium nitrite (Figure 2d) at the center of the star shaped channels. An array of circular channels was designed to demonstrate testing of ascorbic acid, nitrite, and glucose. For ascorbic acid test, the reagents were casted on the circular channels sequentially and dried at room temperature. For example, 0.5 μL of potassium iodide was introduced after drying 0.5 μL of potassium iodate followed by addition of 1 μL of starch reagent. After this, one drop of 1 μL of ascorbic acid was introduced. For nitrite test, one drop of 1.5 μL of Griess reagent was introduced in the circular channels. For making brighter colors multiple drops of Griess reagent maybe added. Upon drying, one drop of 1 μL of sodium nitrite was dropped. For glucose test, one drop of 1 μL of 1.2 M potassium iodide was spotted, followed by spotting one drop of 1 μL of horseradish peroxidase: glucose oxidase (1:5 ratio) on the circular area. Upon drying, one drop of 1 μL of glucose was dropped. The limit of detection (LOD) was projected from the image sets of arrays with varying concentrations for ascorbic acid from 0–0.11 mM, nitrite from 0–1.4 mM, and glucose from 0–100 mM, respectively. The lowest analyte concentration at which a visible color change can be observed and after which no more changes could be seen was considered as the detection limit [22,23]. For citric acid assessment, a ten-star shaped channel was designed as shown in Figure 3b. The outer circles (detection zone) were spotted with 1 μL of phenolphthalein, and the middle circles were spotted with various concentrations of 1 μL of sodium hydroxide. After drying 10 μL of 4 mM citric acid was introduced in the center circle. All the experiments were performed in triplicates at room temperature. The Supplementary Information document explains the detailed reaction mechanism of action for each reagent.

### 2.3. Design of Paper Microfluidics to Identify Biochemical Compounds in Packaged Fruit Juices 

A circular channel was designed for testing the presence of lactose, a biochemical compound found commonly in most packaged fruit juices, fruit flavored milk or dairy based fruit blends. For this, lactaid capsules was powdered and dissolved with whole milk (to make two batches each with 0.1 mg/mL concentration) and filtered using a 0.25 µm PTFE syringe filter. One batch was heated at 100 °C and both batches were assessed by glucose testing, as mentioned previously. In another test, the fruit juice spoilage was assessed by testing for the presence of guaiacol, a biochemical product of spoiled fruit juices. For this, one drop of 1 μL of 30% hydrogen peroxide was spotted on the circular area of the channel, followed by spotting one drop of 1 μL of horseradish peroxidase. Upon drying, one drop of 1 μL of tainted fruit juice was spotted and observed for colorimetric detection.

### 2.4. Designing Multiplexed Test Strips 

A ten-star channel was designed to demonstrate multiplex sensing of the various analytes aforementioned. The designed channel had 3 circles, center circle for introducing the fruit juice, the middle circle for spotting the titrants, and the outer circles for spotting the indicators for colorimetric detection. This was demonstrated with addition of milk and lemonade to the test strip.

### 2.5. UV-VIS Spectrophotometry 

An ultraviolet visible (UV-VIS) spectrophotometer (Agilent Technologies, 8453, Santa Clara, CA, USA) was utilized to gather the absorbance profile and deduce the standard curves for the four main components of fruit juices used for the titration study namely, ascorbic acid, citric acid, nitrite, and glucose. 

## 3. Results and Discussion

### 3.1. Titration on Paper Microfluidics 

In analytical chemistry laboratories, pH indicators are commonly used to determine the endpoint of the reaction or in redox titration experiments. As shown in Figure 2, the pH sensing zones in the six-star channel were covered with pH indicators in different pH range namely; phenol red (from pH 3–10), bromocrescol green (from pH 3–7.5), chlorophenol red (from pH 3–9), and the four-star channel with bromophenol blue (from pH 3–7) respectively. This range was chosen subjective to the pH range of usual fruit juices. These pH indicators exhibited intermediate color change with increment of each pH point within their individual transition range [24]. Hence, four indicators were chosen for cross verification and also to avoid imprecise readings between close pH values. As shown in Figure 2, phenol red is yellow at pH 3 and becomes deeper pink until pH 10, bromocrescol green is yellow at pH 3 and becomes green until pH 7.5, chlorophenol red is pale purple at pH 3 and becomes deep purple until pH 9, while bromophenol blue is yellow at pH 3 and becomes deep blue until pH 7. Phenol red, bromophenol blue, and chlorophenol red offers a large pH range and hence can be used for dynamic pH based applications. The reaction of citric acid (pH 2.97) and sodium nitrite (pH 8.64) with the different pH indicators in varying pH range resulted in three outcomes; (1) neutralization with the near same pH points, (2) decrease in the higher pH points, and (3) increase in the lower pH points, resulting in a visible color change as observed in Figure 2c,d. Figure 2e shows the RGB value for each of the pH indicators from pH 3–10. The goal was to evaluate the pH test using an acid and a base, hence citric acid and sodium nitrite was used. Furthermore, citric acid is commonly found in most fruit juices, and sodium nitrite is mostly used as a food preservative hence, it was appropriate for the study. 

Some common analytes found in most fruit juices include vitamin C (ascorbic acid), citric acid (in most citrus fruit blends), added sugars or sweeteners (glucose), and preservatives (nitrites). Vitamin C was determined by redox titration of potassium iodate with potassium iodide, such that the free iodine formed reacted with starch indicator once the ascorbic acid was completely oxidized forming a dark black-purple complex [25]. Glucose detection was based on the oxidation-reduction reaction to produce a colored complex that could be identified by the naked eye. The colorimetric assessment involved interaction of glucose with glucose oxidase yielding hydrogen peroxide, which was further reduced to water by horseradish peroxidase. The potassium iodide was oxidized to iodine generating the brown colored complex [26]. Griess reagent is commonly used for detection of nitrites. This reagent consists of an aromatic amine sulphanilamide which reacts with nitrite to form a diazonium salt, which then combines with N-(1-naphthyl)-ethylenediamine dihydrochloride (NEDD) to form a pink colored azo dye [27]. Citric acid assessment was based on acid-base titration [21], such that the reaction of citric acid with sodium hydroxide reaches an equivalence point, else it climbs above this point (Figure 3b). For ascorbic acid, the LOD was found to be 1.47 µM with a linear range from 1–20 µM (Figure 3a), for glucose LOD was 20 mM with a linear range from 10–40 mM (Figure 3d), and for nitrite LOD was 0.06 mM with a linear range from 0.30–1.40 mM (Figure 3c). This LOD is sufficient to the concentration expected in commercially available beverages. As summarized in Table 1, the concentration of the analytes found in the listed beverages are at a higher concentration than the LOD achieved in the current study, making our multiplexed µPADs, applicable for identification of analytes in the beverages. The intensity expressed at y-scale in Figure 3e–g is the RGB intensity of the images with integer values from 0–255 acquired using ImageJ software [28]. ImageJ software was used for selecting the Region of Interest (ROI) by thresholding (or segmentation) to avoid mistakes in prediction due to uneven color distribution. A detailed explanation is provided in the Supplementary Information.

The analytes identified in our study are suitable for on-site detection from fresh or packaged fruit juices by building easily available premade tests strips spotted with the necessary titrants and the indicators.

### 3.2. Identifying Biochemical Compounds in Packaged Fruit Juices

**Figure 4:**
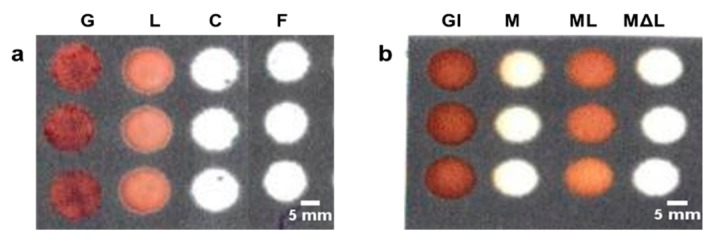
Testing biochemical compounds found in packaged fruit juices. (**a**) Testing of lactose by adding lactaid in milk; (**b**) testing fruit juice spoilage. GU: guaiacol; S: spoiled lemonade; C: control; F: fresh lemonade; Gl: glucose; M: milk; ML: milk + lactose; MΔL: milk + heated lactose. (Scale bar: 3 mm).

In the present study, we identified two common biochemical compounds namely, lactose, mostly found in fruit flavored dairy products, and guaiacol, a non-pathogenic biochemical product of tainted fruit juices. To identify lactose found in common dairy based fruit drinks, lactaid capsules were added to the milk to supply the enzyme lactase which converted lactose to glucose. The glucose assessment was done as mentioned previously, and displayed a dark brown color. Milk added with lactaid capsules showed a light brown color, while milk with no lactaid and lactiad added milk, but heated to ~70 °C (to denature the lactase enzyme) displayed no color (Figure 4a).

Since raw milk contains lactose, the glucose oxidase in the absence of glucose did not undergo any reaction and hence displayed no color. The lactiad contained milk after heating, suffered from enzyme degradation and hence had no converted glucose to initiate the reaction. Another biochemical product guaiacol found in spoiled fruit juices was also identified in a separate test. Guaiacol is a non-pathogenic chemical that is produced by the alicyclobacillus bacterial strains found in spoiled or tainted fruit juices [34]. In presence of guaiacol, the hydrogen peroxide and peroxidase reacted to form tetra guaiacol a visible brown colored complex (Figure 4b). The identification of guaiacol could help determine the spoilage/freshness of the fruit juice.

### 3.3. Multiplexed Test Strips to Identify the Components of Fruit Juices

**Figure 5:**
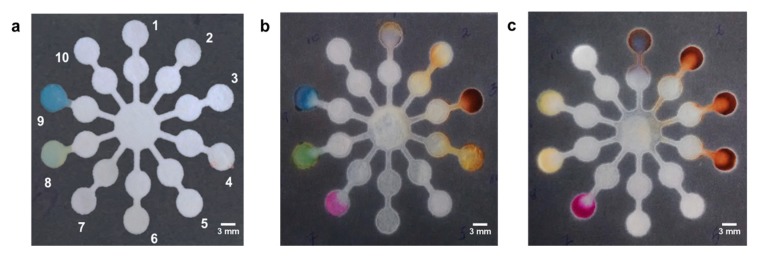
Multiplex paper microfluidic setup for testing several analytes. (**a**) After adding reagents; (**b**) after adding milk; (**c**) after adding lemonade. 1: ascorbic acid; 2: glucose; 3: lactose; 4: heated lactose; 5: nitrite; 6: guaiacol; 7: citric acid; 8: bromocresol green (7.53); 9: bromophenol blue (pH = 7.12); 10: control. (Scale bar: 3 mm).

Multiplex sensing is the ability to detect multiple analytes on a single platform. The key advantages of multiplex sensing are ease of use, flexibility, low cost, and low sample volume. Multiplex sensing was demonstrated on the final premade test strip, after loading the necessary titrants and indicators as labelled in Figure 5a.

After adding milk blend, labels 3, 7, 8, and 9 showed visible color change. From Figure 5b, it can be concluded that the milk contained lactose, and had a pH of around 6.8–7. Label 8, revealed the citric nature of the milk bend. Figure 5c, highlighted labels 1, 2, 3, 4, 7, 8, and 9 after introducing lemonade. From the labels, it was concluded that the lemonade had vitamin C (label 1), contained a lot of added sugars (labels 2, 3, 4), was citrus and the pH was in the range of 4–5. Once the titrants and indicators were spotted on the test strips, it took only 45–60 s to generate color at the detection zone. In order to avoid any false positive results, we optimized the volume/area ratio of all the channel designs and have also tested for any interferences from matrix or color pigments. (Supplementary Information). Such paper based prefabricated tests strips can allow detection of multiple analytes of interest, and provide useful information about the ingredients on the packaged fruit juice, to help know your drink, and make a healthy beverage choice.

### 3.4. UV Spectrophotometry

**Figure 6:**
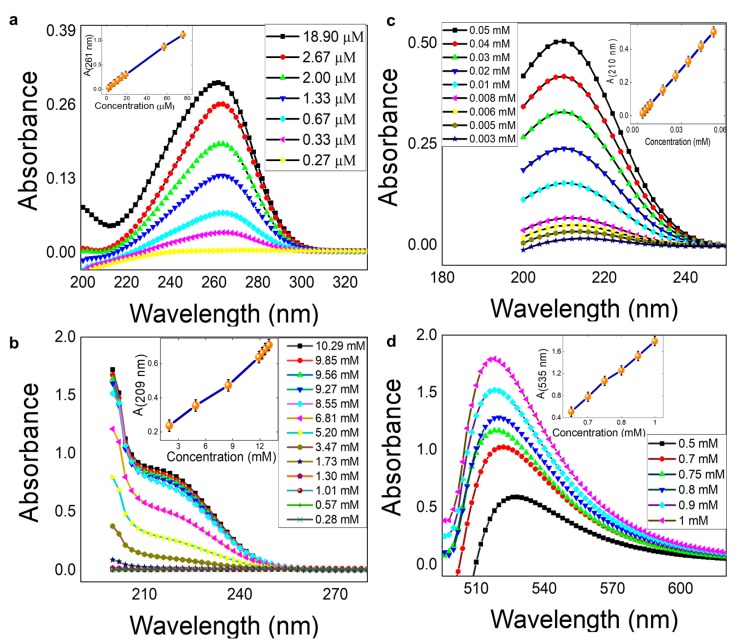
Absorbance Profile and Standard Curve. (**a**) Ascorbic acid; (**b**) nitrite; (**c**) citric acid; (**d**) glucose.

The absorbance profile and the standard curves for ascorbic acid (~λ_261_) (Figure 6a), nitrite (~λ_209_) (Figure 6b), citric acid (~λ_210_) (Figure 6c), and glucose (~λ_535_) (Figure 6d), was successfully obtained using a UV-VIS spectrophotometer. The spectrophotometric data acquired was used to correlate the results obtained from paper microfluidics. Although the goal of the present study was chemical based colorimetric detection of analytes, with a Yes/No response, the calibration curves can be helpful for determining the unknown quantitative concentration and validation of the results obtained from paper microfluidics. Also, it can be used to explain why the particular chemical is colored in that particular way before/after the reactions. Comparison of the LOD and linear range of μPADs with traditional absorbance spectrophotometry method is detailed in Supplementary Information.

## 4. Conclusions

The goal of this hands-on approach experiment was to apply the analytical chemistry concepts to real world applications using a simple paper microfluidic technology. In this study, we demonstrated detection of pH, vitamin C, sugars, preservatives, milk allergen, and the biochemical compound responsible for food spoilage found in packaged fruit juices using paper microfluidics. Further, we demonstrated an inexpensive and easy way of converting paper-based analytical devices for food safety applications. The fabrication of the sensor and the detection of the analytes can be performed easily in resource-poor settings. The current research could be upgraded for on-spot detection by producing premade tests strips, and for several other packaged food items. In conclusion, paper microfluidics can be utilized as multiplexed sensors to perform serval analytical chemistry tests on a single platform with good sensitivity, reproducibility, and detection limit. The future work should be focused towards integrating paper microfluidics with automated readers for food safety and monitoring applications.

## Figures and Tables

**Table 1:** Summary of the concentration of common analytes present in beverages.

Analyte	Common Beverages	Amount Present	Concentration (C)	Present Study (LOD)	C/LOD	Refs.
Vitamin C	Low citric fruit juice	23–66 mg/125 mL	1–3 mM	1.47 µM	~1000	[29]
	Fruit & Vegetable mixed juice	35–73 mg/12 mL	16–34 mM	1.47 µM	~1000	
Nitrite	Fruit & Vegetable juice	0.71 mg/100 g	0.1 mM	0.06 mM	1.5	[30,31]
Sugar	Sodas, Fruit juice, Ice Tea	11 g/100 mL	600 mM	20 mM	30	[32,33]

## Supplementary Materials

The following are available online at https://www.mdpi.com/1424-8220/19/6/1286/s1, Experiment protocol, Mechanism of the reaction, Table S1: Description of the pH indicator and their pH range, Table S2: Description of the Assays performed, Table S3: Summary of detection strategy.
