# Supplementary material for: Multiplexed Paper Microfluidics for Titration and Detection of Ingredients in Beverages
Source: Sensors (Basel). 2019 Mar 14;19(6):1286. doi: 10.3390/s19061286 (PMC6471555; doi:10.3390/s19061286)
Supplement: Supplementary file 1 [file sensors-19-01286-s001.pdf]

Supporting Information

Multiplexed Paper Microfluidics for Titration and Detection of Ingredients in Beverages

Alisha Prasad[1], Tiffany Tran[2], Manas Ranjan Gartia[1*]

[1]Department of Mechanical and Industrial Engineering, Louisiana State University, Baton Rouge, LA 70803

[2]St.Jospeh's Academy, 3015 Broussard St, Baton Rouge, LA 70808

*Corresponding author: mgartia@lsu.edu

Table of Content

Section. S-1 Testing matrix and color pigment interferences …………………………………..……..….. S-2

Section. S-2 Demonstration of selecting Region of Interest in ImageJ…………….…………...……...….. S-3

Section. S-2 Preparation of reagents……………………………………………………………...……….. S-4

Section. S-3 Mechanism of the assays performed ……………………………………………….…… S-5

Ascorbic acid Test……………………………………………………………………………….. S-5

Nitrite Test……………………………………………….……………………………………..… S-6

Citric acid Test………………….………………………….…………………...……………….…. S-7

Glucose Test…………………………………………….……………………….……………..… S-8

Lactose Test……………………………………….…..…………………………………….….… S-8

Fruit Juice Spoilage Test ……………………………….……………..…………………...…… S-9

Table. S-1 Description of the pH indicator and their pH range …….…………………….....…………… S-9

Table. S-2 Description of the Assays performed ……………………………………………...…...…. S-9

Table. S-3 Summary of the Detection Strategies ………………………………………….…….……. S-10

Table S4: Comparison of LOD and linear range of μPADs with traditional absorbance spectroscopy method ………………………………………….……………………………………….…….……. S-12

Section. S1: Testing matrix interferences: For our paper-based microfluidic systems (μPADs), we used Whatman No.1 filter paper (11 μm pore size) (Bio rad Laboratories, USA). Wax was printed to make the hydrophobic part of the sensor. We investigated the possible interferences/matrix effect due to color pigments in the sample, or possible formation of complex or precipitates due to reaction among analytes. The results are presented in Figure R1.

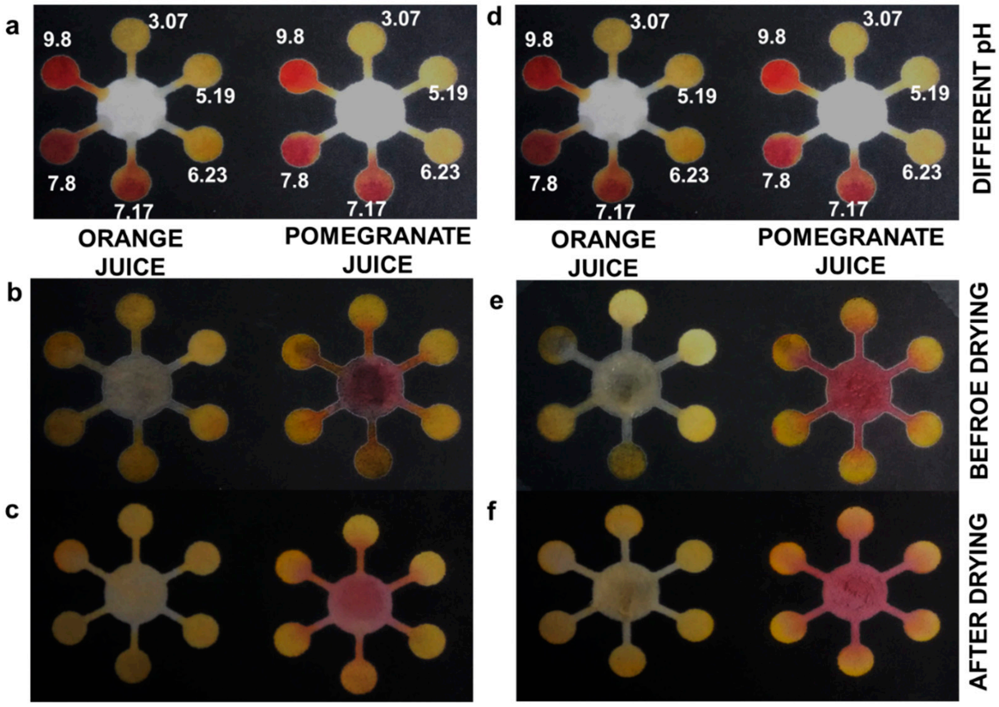

Fig R1. Testing matrix interferences by evaluating pH test using different beverages (a) Phenol red at different pH (b) After adding 10μL of juice (c) After drying; (d) Phenol red at different pH (e) After adding 20μL of juice (f) After drying; Orange juice (pH:3.99), Pomegranate juice (pH:3.63).

In order to avoid any false positive results, we optimized the volume/area ratio of all the channel designs. For the six-star channel, the outer circles (or the test zones; ø=3 mm) required 1.5 μL of fluid to cover (wet) the area, while the middle circle (or the sample zone; ø=8 mm) required 10 μL of fluid to reach the tests zones. This optimization is important to control the volume of fluid flow in μPADs[1]. It is important to note that this might differ according to the channel design, choice of paper, and amount of wax used. **Fig R1** shows the matrix interference based on a simple pH test using different beverages. **Fig R1 (b)** shows that after adding 10μL of juices, all the test zones displayed an even yellow color tone. On the other hand, **Fig R1 (e),** showed that when 20μL of juices was used, it displayed an uneven yellow color tone, indicating

possible matrix interference. These results support the importance of volume/area ratio in μPADs. In order to strengthen the colorimetric interpretation, and avoid any false positive results we have included a series of pH indicators namely, phenol red, bromophenol blue, chlorophenol red, and bromocrescol green at wide pH ranges, and presented a pH chart to correlate and validate any tests.

Testing interferences due to color pigments:

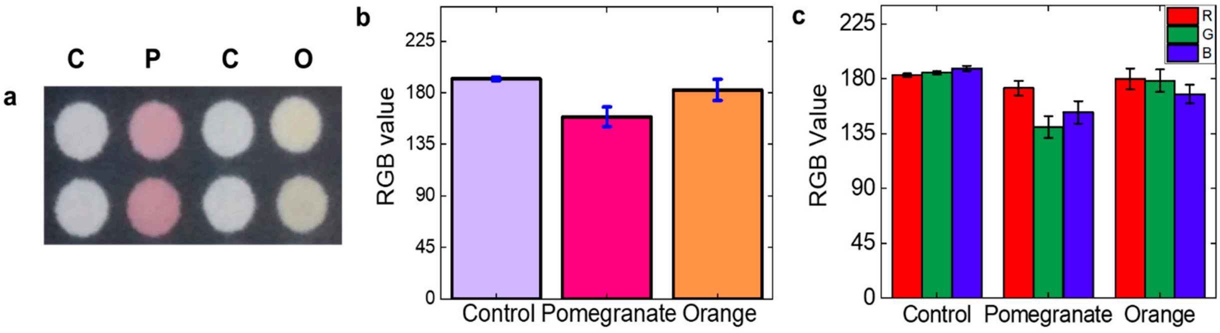

Fig R2. Testing interferences due to color pigments. (a) Loading different beverages on the μPADs (b) Comparing the RGB intensity value. C: Control (Distilled Water), P: Pomegranate Juice; O: Orange Juice.

To test interferences due to color pigments from samples we introduced 2 mL of commercially available colored beverage namely, pomegranate juice (P) and orange juice (O) in its real state without any dilution or addition of any reagents or indicators to check differences in the RGB value. Distilled water (C) was used as a control. As seen from the RGB value in **Fig R2 (b),** the color pigments can lead to interferences. Furthermore, as shown in **Fig R2 (c)**, the interference can be monitored by checking the individual RGB value, wherein the Red Channel in the Pomegranate juice shows more contribution. The interpretation of false results can be avoided by following the protocol as mentioned above.

Section. S-2 Demonstration of selecting Region of Interest in ImageJ

The ImageJ software enables separating pixels which fall within a desired range of intensity values (Region of Interest (ROI)) from those which do not, by thresholding (or segmentation). Thresholding is an effective method to measure complex or non-uniform features in an image. The ROI in each image contains same number of pixels. The ROI approach avoids mistake in prediction due to uneven color distribution as shown in **Fig R3**. The *Analyze* command in ImageJ software is used to count and measure the thresholded images. The *Analyze* menu also contains a *Set Measurements* dialog box, where in the user can obtain information about the area of selection in pixels, mean gray value, and integrated density *etc*. Formula for

calculating the RGB/pixel value is = $\left(\frac{\sum_i x_i\, I_i}{\sum_i x_i}\right)$; where x_i is the total number of colors and I_i is the intensity of each color.

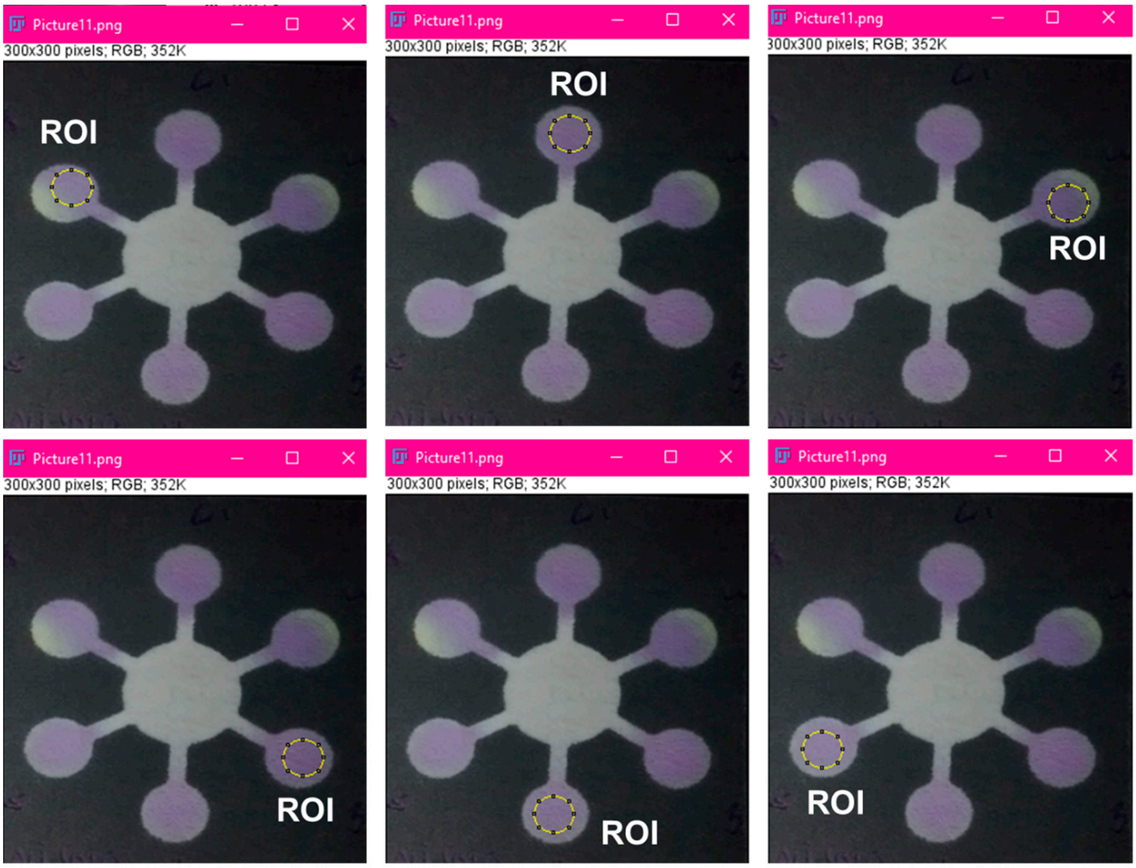

Fig R3. Demonstration of selecting Region of Interest (ROI) in ImageJ.

Section. S2: Preparation of reagents

- Phenol Red (1.77 mg phenol red, 8.2 mg CTAB, and 5 µL of 1M NaOH was dissolved in 5 mL distilled water)
- Bromophenol Blue (0.1 g was dissolved in 100 mL of methanol)
- Bromocrescol Green (0.1 g was dissolved in 100 mL of ethanol)
- Chlorophenol Red (3. 17 mg chlorophenol red and 10.93 mg CTAB, was dissolved in 5 mL distilled water)
- Phenol Red (from pH 3-10), Bromocrescol Green (from pH 3-7.5), Chlorophenol Red (from pH 3-9), and the four-star channel with Bromophenol Blue (from pH 3-7), was prepared by adjusting the pH by adding 1 µL of 1N NaOH (for basic pH), or 1.5 M of HCl (for acidic pH), slowly dropwise, and checking the pH using a pH meter (HI5221, Hannah Instruments, Rhode Island, USA).
- Ascorbic acid (For 0.11 mM, 1 mg was dissolved in 50 mL distilled water)
- Sodium nitrite (For 1.44 mM, 10 mg was dissolved in 100 mL distilled water)

- Glucose (For 100mM, 1.8 g was dissolved in 100 mL distilled water)
- Citric Acid (For 4 mM 71 mg in 100 mL distilled water)
- Sodium Carbonate (For 0.1 M, 0.10 g was dissolved in 10 mL distilled water)
- Potassium Iodide (For 1.2 M, 0.99 g was dissolved in 5 mL distilled water)
- Potassium Iodate (For 2 mM, 4.3 mg was dissolved in 10 mL distilled water)
- Sodium Hydroxide (For 1M, 0.4 g was dissolved in 10 mL distilled water)
- Lactaid Capsules (For 0.1 mg/mL, 1 capsule of 1 mg was dissolved in 10 mL milk)
- Horseradish peroxidase: Glucose oxidase (Final solution was made by suspending them in1:5 ratio by weight)

Section. S3: Mechanism of the assays performed

Ascorbic acid Test:

The detection of ascorbic acid/Vitamin C is based on the redox titration of potassium iodate with potassium iodide to form reduced iodine as shown in the first equation below. Second equation shows that this free iodine formed oxidized the ascorbic acid to dehydroascorbic acid and is reduced to iodide ions. Once the ascorbic acid was completely oxidized the free iodine (I^-) reacts with the starch indicator forming a dark black-purple complex.

$$\underset{\text{Potassium iodate}}{KIO_3^-} + \underset{\text{Potassium iodide}}{KI} + 12H^+ \longrightarrow 6I_2 + 6H_2O$$

$$I_2 + \underset{\text{Ascorbic acid}}{C_6H_8O_6} \longrightarrow \underset{\text{Dehydroascorbic acid}}{C_6H_6O_6} + 2H^+ + 2I^-$$

Nitrite Test:

The detection of nitrites is on based on the Griess reagent. This reagent is made of 2% of an aromatic amine sulphanilamide in 5% phosphoric acid, and 0.2% N-(1-naphthyl)-ethylenediamine dihydrochloride (NEDD). In presence of nitrite (NO^{2-}), the aromatic amine reacts to form a diazonium salt, which then reacts with NEDD to form a pink colored azo dye as shown in the equation below. The mechanism of Griess assay is based on the chemical diazotization reaction.

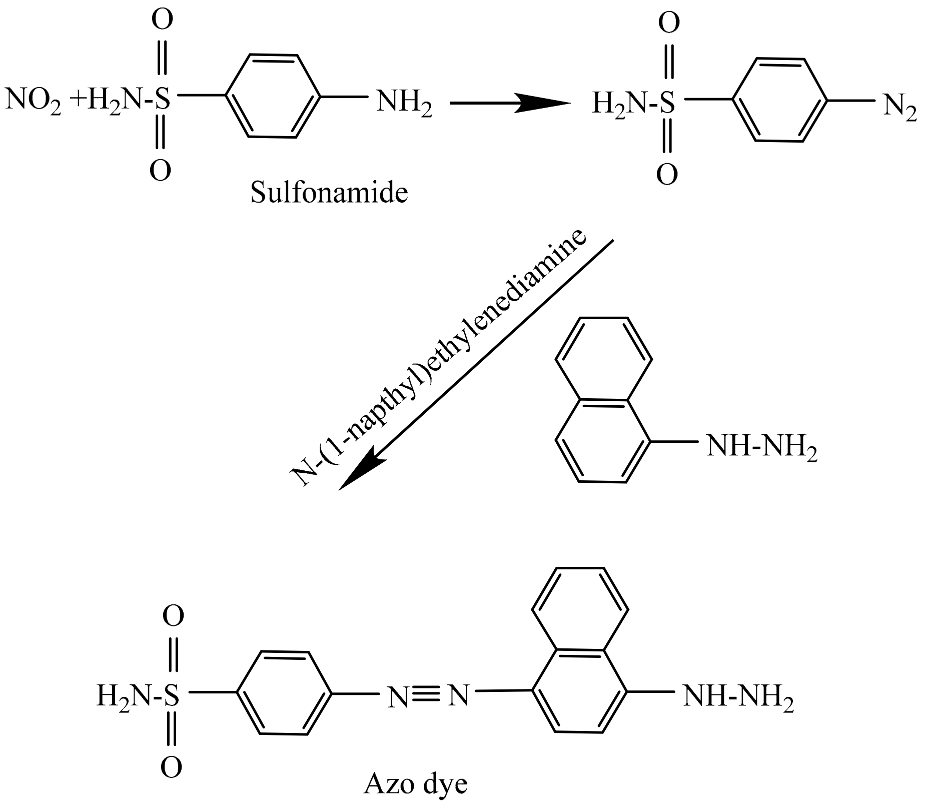

Citric acid Test:

Citric acid assessment was based on acid-base titration such that the reaction of citric acid with sodium hydroxide reaches a neutralization point. Citric acid is basically a triprotic acid with three carboxylic acid groups, three ionizable acidic hydrogen atoms and three pKa values as shown below. The mechanism is that upon interaction of citric acid with a strong base, the citric acid, crosses a buffer region such that it's pH climbs slowly then more sharply. For example, addition of one drop of 0.1 M NaOH climbs the pH slightly below 7, and upon addition of another one drop after the equivalence point the pH of the titration spikes between 7 to above 9. Phenolphthalein a visual acid-base indicator was used to observe the change from colorless to pink.

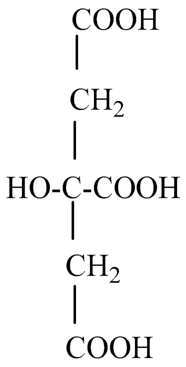

Citric acid

$$3NaOH + H_3C_6H_30_7 \longrightarrow Na_2C_6H_3O_7 + 3H_2O$$

Sodium hydroxide — Citric acid — Sodium citrate

Glucose Test:

The detection of glucose was based on the enzyme mediated oxidation-reduction reaction. As shown in the first equation below, glucose was oxidized with glucose oxidase yielding hydrogen peroxide. In the second step, hydrogen peroxide was reduced by horseradish peroxidase such that the potassium iodide was oxidized to iodine generating a brown colored complex. The horseradish peroxidase is a hydrogen peroxide (H_2O_2) decomposing enzyme that acts as a catalyst to oxidize phenolic/non- phenolic substrates (or -RH) and amplifies the signals for the detection of desired targets. The reaction is as follows: $2RH + H_2O_2 \rightarrow 2R^{\circ} + 2H_2O$.

$$\underset{\text{Glucose}}{C_6H_{12}O_6} + H_2O + O_2 \xrightarrow{\text{Glucose oxidase}} \underset{\text{Gluconic acid}}{C_6H_{12}O_7} + \underset{\text{Hydrogen peroxide}}{H_2O_2}$$

$$H_2O_2 + \underset{\text{Potassium iodide}}{2KI} \xrightarrow{\text{Horseradish peroxidase}} 2KOH + \underset{\text{Brown color}}{I_2}$$

Lactose Test:

The detection of lactose was based on the indirect glucose assessment. Lactose was first converted to glucose by the enzyme lactase as shown in the equation below. The converted glucose was assessed by glucose assay as mentioned previously.

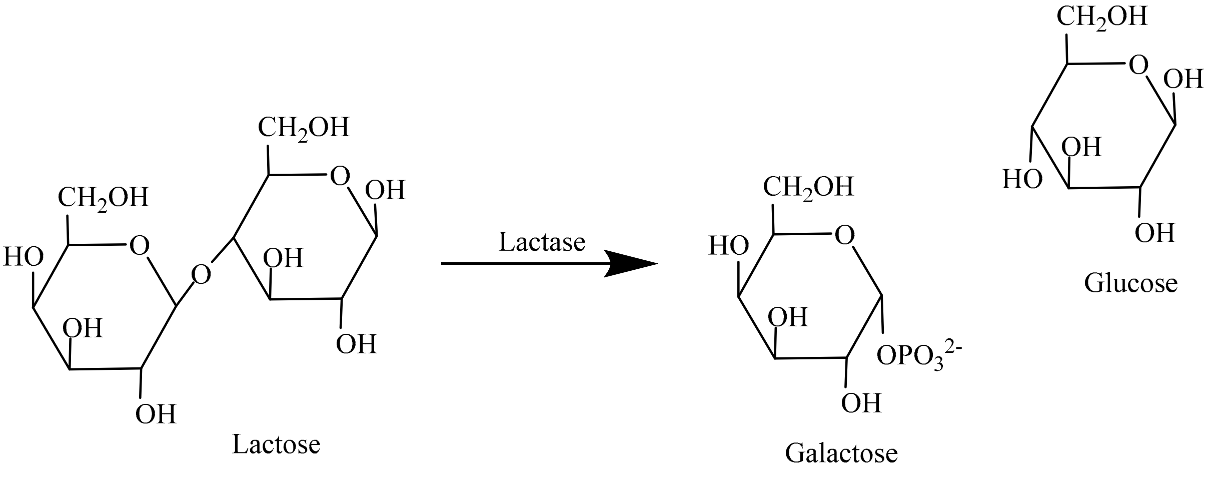

Fruit Juice Spoilage Test:

The fruit juice spoilage test was based on the guaiacol detection. Tainted fruit juices commonly get invaded with *alicyclobacillus* bacteria population. These strains produce a non-pathogenic chemical guaiacol. In presence of guaiacol, the hydrogen peroxide and peroxidase reacted to form tetra guaiacol a brown colored complex.

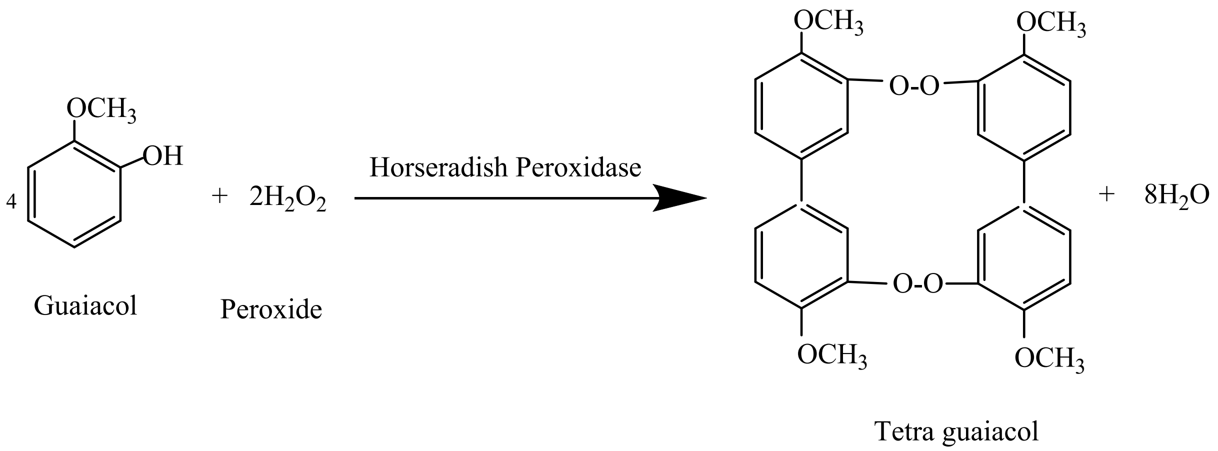

Table S1: Description of the pH indicator and their pH range

pH indicator	Low pH color	High pH color
Phenol Red	6.4	8.0
Bromophenol Blue	3.0	4.6
Bromocrescol Green	3.8	5.4
Chlorophenol Red	4.8	6.7

Table S2: Description of the Assays performed

Assays	Mechanism	Analyte Sensed	Limit of Detection
pH	Acid-base titration	Citric acid (pH 3-4.5)	-
		Phenol red (pH 3-10)	-
		Bromophenol blue (pH 3-7)	-
		Bromocrescol green (pH 3-7.5)	-
		Chlorophenol red (pH 3-9)	-
Vitamin C titration	Oxidation-reduction	Ascorbic acid	1.47 μM
Glucose Assay	Enzyme based Oxidation-reduction	Glucose	20 mM
Griess Assay	Diazotization reaction	Nitrite	0.06 mM

Glucose Assay	Enzyme based Oxidation-reduction	Lactose converted to Glucose	20 mM
Guaiacol Assay	Enzyme based Oxidation-reduction	*Alicyclobacillus* bacterial strains	-

Table S3: Summary of the Detection Strategies

Analyte Detected	Detection Strategies	Detection Principle	Reagents used	Dynamic range	Advantages	Disadvantages	Refs.
Glucose	μPADs-by Wax Jetting of filter paper	Colorimetric	Glucose oxidase, horseradish peroxidase Potassium iodide	0-50 mM	low-cost, simple, easy-to-use and fast fabrication	The 3D Wax Extruder requires to be heated, and run on specific voltages depending on the thickness of printed wax.	[2]
	μPADs-by silanization and wet etching of hydrophilic filter paper	Colorimetric	Glucose oxidase, horseradish peroxidase, Potassium iodide	0-20 mM	equipment free, requires no metal mask	Expensive patterning and etching agents. Requires skilled personnel	[3]
	μPADs-by using wax printer	Colorimetric	Glucose oxidase, horseradish peroxidase, Potassium iodide	0-100 mM	No functionalization or chemical modification of the filter paper use	Higher detection limit	Present work
pH	Poly(dimethyl siloxane) (PDMS) barriers	Colorimetric	Bromothymol Blue	pH 6.5-8.0	PDMS is non-toxic	Poor resolution of printing i.e. minimum 1	[4]

	used to create channels onto filter paper					mm size attainable	
	PDMS stamping to create hydropho bic channels	Colorimetr ic	Phenol red, Chlorophenol red	pH: 4.0-9.0	Stable at each pH range	Low Dynamic range	[5]
	µPADs-by using wax printer	Colorimetr ic	Phenol red, Bromophenol blue, Bromocresol green, Chlorophenol red	pH 3.0-10.0	Stable at each pH range	Higher Dynamic range	Prese nt work
Nitrite	PDMS stamping to create hydropho bic channels	Colorimetr ic	Nafion, 2-propanol, polyethylene glycol 400, citric acid, Sulphanilamid e, N-1-Naphthyl ethylenediami ne dihydrochlorid e	Not mentio ned	Stable, and low detection limit attained	Reagents used can be allergic	[5]
	PMMA use to create hydropho bic templates on filter paper	Colorimetr ic	Benzenesulfon amide, N-(1-naphthyl) ethylenediami ne, citric acid, methanol	0.0780–2.50 mM	Simple and easy to use	p-Amino benzenesulfon amide can lead to inflammation and Methanol is highly volatile	[6]
	µPADs-by using wax printer	Colorimetr ic	Griess reagent	0-1.4 mM	Stable	Simple one step process	Prese nt work
Ascor bic acid	By used wax printed and functionali zion by	Electroche mical	potassium ferricyanide	0.001 - 0.5 M	Good ion exchange	Applicable for Ascorbic acid at higher concentrations	[7]

	sputter a gold mask						
	μPADs-by using wax printer	Colorimetr ic	Starch indicator, Potassium iodide, potassium iodate	0-0.11mM	Simple	Starch indicator contains mercury which can be toxic	Prese nt work

Table S4: Comparison of LOD and linear range of μPADs with traditional absorbance spectroscopy method

**Analyte Sensed**	**Linear range**		**Limit of Detection**	
	μPADs	**Traditional**	μPADs	**Traditional**
Ascorbic acid	1-20 μM	0.67-18.90 μM	1.47 μM	2.00 μM
Glucose	10-40 mM	0.50-1.00 mM	20 mM	0.70 mM
Nitrite	0.30-1.40 mM	9-14 mM	0.06 mM	3.47 mM

The comparison of LOD and linear range of μPADs with traditional absorbance spectroscopy method is presented above. From the results tabulated **(Table S4)** it was observed that ascorbic acid and glucose had a wide linear range whereas nitrite had a narrow linear range in comparison to the traditional approach. The LOD for μPADs based assay for ascorbic acid was one order of magnitude lower than the traditional assay, and for nitrite it was 50-times lower than the traditional assay. However, the LOD for glucose was found to be 20-times higher than the traditional assay. This is because our aim was to develop a simple paper based colorimetric detection system that is capable of distinguishing multiple analytes simultaneously by naked eyes. In the current study, the common analytes identified are present in beverages in much higher concentrations than the achieved detection limit, making our multiplexed μPADs applicable to this specific task. Furthermore, in the current settings we performed all the study on the Whatman filter paper Grade 1, without any surface treatment procedures to make it a low-cost, easy-to-use approach for conducting the titration based assays on-site.

References

1. Prasad, A.; Hasan, S.M.A.; Grouchy, S.; Gartia, M.R. DNA microarray analysis using a smartphone to detect the BRCA-1 gene. *Analyst* **2018**.
2. Yang, J.; Zhu, L.; Tang, W. Fabrication of paper micro-devices with wax jetting. *RSC Advances* **2016**, *6*, 17921-17928.

3. Cai, L.; Xu, C.; Lin, S.; Luo, J.; Wu, M.; Yang, F. A simple paper-based sensor fabricated by selective wet etching of silanized filter paper using a paper mask. *Biomicrofluidics* **2014**, *8*, 056504.
4. Bruzewicz, D.A.; Reches, M.; Whitesides, G.M. Low-cost printing of poly (dimethylsiloxane) barriers to define microchannels in paper. *Analytical chemistry* **2008**, *80*, 3387-3392.
5. Lopez-Ruiz, N.; Curto, V.F.; Erenas, M.M.; Benito-Lopez, F.; Diamond, D.; Palma, A.J.; Capitan-Vallvey, L.F. Smartphone-based simultaneous pH and nitrite colorimetric determination for paper microfluidic devices. *Analytical chemistry* **2014**, *86*, 9554-9562.
6. Wang, B.; Lin, Z.; Wang, M. Fabrication of a paper-based microfluidic device to readily determine nitrite ion concentration by simple colorimetric assay. *Journal of Chemical Education* **2015**, *92*, 733-736.
7. Murphy, A.; Gorey, B.; De Guzman, K.; Kelly, N.; Nesterenko, E.; Morrin, A. Microfluidic paper analytical device for the chromatographic separation of ascorbic acid and dopamine. *RSC Advances* **2015**, *5*, 93162-93169.
